# African swine fever virus P72 genotyping tool

**DOI:** 10.1128/mra.00891-23

**Published:** 2024-01-08

**Authors:** Mark Dinhobl, Edward Spinard, Hillary Birtley, Nicolas Tesler, Manuel V. Borca, Douglas P. Gladue

**Affiliations:** 1 U.S. Department of Agriculture, Agricultural Research Service, Foreign Animal Disease Research Unit, National Bio and Agro-Defense Facility, Manhattan, Kansas, USA; 2 U.S. Department of Agriculture, Agricultural Research Service, Foreign Animal Disease Research Unit, Plum Island Animal Disease Center, Orient, New York, USA; 3 Center of Excellence for African swine fever Genomics, Guilford, Connecticut, USA; 4 Oak Ridge Institute for Science and Education (ORISE), Oak Ridge, Tennessee, USA; DOE Joint Genome Institute, Berkeley, California, USA

**Keywords:** ASFV, African swine fever, ASF, African swine fever virus, P72, tool, genotyping, swine

## Abstract

Historically, genotyping of African swine fever virus was based on partial sequencing of B646L (p72). Until recently, the number of differences that defined genotypes was ambiguous. This tool allows a sequence to be uploaded and will report its closest matches along with its likely p72 genotype.

## ANNOUNCEMENT

P72 is an essential major capsid protein for the causative agent of African swine fever, African swine fever virus (ASFV), and historical genotyping has been based on differences in its nucleotide coding sequence since it is relatively conserved (1). Still, ASFV is a large double-stranded DNA virus that encodes 160–190 genes, and genetic differences between isolates often arise from deletions and fusions outside of p72, such as within the multi-gene family genes. While genotyping by p72 alone cannot fully elucidate the number of different isolates in each region, it remains the most available and economical method for disease tracking in many of the regions in which ASFV causes outbreaks. Previously, the number of different genotypes was considered to be 25; however, the parameters for defining a genotype were never formally established for ASFV. We recently performed a reanalysis of p72 genotypes and determined there are only 20 distinct full-length p72 protein sequences that can be classified within five different genotypes (Genotypes 1, 2, 8, 9, and 23) using a simple cutoff of three amino acid differences (2).

Here we present a new tool that will allow researchers to quickly and simply identify the p72 genotype of newly sequenced full-length genome or p72 sequences. This tool is available through the source code GitHub or through using the website https://ASFVgenomics.com/upload. On either, users can submit either a nucleotide or amino acid sequence of ASFV. The sequence is run through a BLAST script (3) that searches against a curated database based on the 20 distinct sequences (Table 1) using the following parameters: minimum *E* value = 0.001, gap opening penalty = 11 (default), and gap extension penalty = 1 (default). After finding the matches, it will then return the closest matched isolate(s)*, the percent identity, and the likely p72 genotype.

**TABLE 1:** Representative isolates from the curated p72 database

Accession number	Isolate	Genotype
AM712239	Benin 97/1	1
AY261362	Mkuzi 1979	1
AY578697	Ker	1
L76727	Dominican Republic 2 (DR2)	1
M34142	BA71V^a^	1
AY578692	E70	1
FR682468	ASFV Georgia 2007/1	2
AY261366	Warthog	2
MN336500	RSA_2_2008	2
MN207061	HBNH-2019	2
OM461370	A9_21_3	2
ON400500	YNFN202103	2
AY261361	Malawi Lil-20/1	8
ON409981	TAN/08/Mazimbu	8
AY261360	Kenya 1950	9
KM111294	Ken05/Tk1	9
MN886937	Kitali	9
L27499	Uganda	9
KT795353	ETH/AA	23
KT795355	ETH/017	23

^a^ Partial p72 sequences or partial matches could equally match multiple p72 genotypes. Accordingly, a warning is provided if the matched p72 sequence is short (less than 630 long) or very short (less than 600 long) to inform the user that there could be matches to multiple possible p72 genotypes and advises the user to look at the raw BLAST output. Tests for speed of the of the program indicate that running a full ASFV genome through the script took 6.48 seconds on a standard laptop (2.4-GHz core processor, 32-GB memory) and less than 2 seconds on the website.

## Data Availability

The source code for the tool is presented at https://github.com/Global-ASFV-Research-Alliance/B646LGenotypingTool, and a website for usage is presented at www.ASFVgenomics.com.
